# Anatomical variations of the external jugular veins and collaterals incidentally diagnosed with computed tomography in Shih Tzu dogs

**DOI:** 10.3389/fvets.2024.1464750

**Published:** 2024-10-17

**Authors:** Seoyoung Cho, Jupill Chang, Woosuk Kim, Kidong Eom, Jaehwan Kim

**Affiliations:** ^1^Department of Veterinary Medical Imaging, College of Veterinary Medicine, Konkuk University, Seoul, Republic of Korea; ^2^SD Animal Medical Center, Seoul, Republic of Korea; ^3^Department of Anatomy, College of Veterinary Medicine, and Veterinary Science Research Institute, Konkuk University, Seoul, Republic of Korea

**Keywords:** Shih Tzu dogs, external jugular vein, variations, computed tomography, vascular

## Abstract

**Introduction:**

The external jugular vein (EJV) is a superficial vein of the neck in dogs; its significance is evident in veterinary clinical practice, encompassing surgeries and interventional procedures. However, there have been no reports on EJV variations in canines, despite extensive studies on variations in the jugular veins in humans. This study aimed to use CT imaging to evaluate the prevalence of anatomic vascular variations of the EJVs in Shih Tzu dogs and to describe the clinical and CT characteristics of these vascular variants.

**Methods:**

This is a retrospective, multi-center study. The medical imaging records of Shih Tzu dogs that underwent pre- and post-contrast CT examinations of the head, neck, and thorax at the Veterinary Medical Teaching Hospital, Konkuk University, and 10 referral hospitals between 2015 and 2023 were reviewed.

**Results:**

We defined five types of EJV vascular variants: normal (type I), unilateral hypoplasia (type II), unilateral aplasia (type III), bilateral hypoplasia (type IV), and bilateral aplasia (type V), based on the morphological and diameter differences observed in the transverse images of Shih Tzu dogs. CT images from 547 Shih Tzu dogs revealed 119 cases (21.7%) of EJV variants. Type I was observed in 428 dogs (78.2%), type II in 46 dogs (8.4%), type III in 41 dogs (7.5%), type IV in 14 dogs (2.6%), and type V in 18 dogs (3.3%). In types II–V, compensatory drainage through the internal jugular vein (IJV) was observed, often involving the medial passage of the maxillary or linguofacial veins. A moderate negative correlation (*R* = −0.5) was recorded between the hypoplastic EJV and the affected-side IJV (*p* < 0.01). Some cases exhibited other supplementary drainage routes, such as the hyoid venous arch or median thyroid vein. Additionally, 63 persistent left cranial vena cava (PLCVC) cases (11.9%) were identified among 529 Shih Tzu dogs, showing a significant association with EJV abnormalities (*p* < 0.05).

**Discussion:**

Overall, this study identified anatomical variants of the EJV in Shih Tzu dogs and introduced a new classification system. These findings revealed that EJV variants and compensatory tributary enlargement were more prevalent than previously recognized, emphasizing the need to consider these nuances in veterinary procedures and imaging.

## 1 Introduction

The external jugular vein (EJV, vena jugularis externa) is a superficial vein of the neck that is mainly formed by the convergence of the linguofacial and maxillary veins (vena linguofacialis and maxillaris) caudal to the mandibular salivary gland (glandula mandibularis) situated between these two veins. The maxillary vein receives venous drainage from the caudal auricular and superficial temporal veins (vena auricularis caudalis and temporalis superficialis). The EJV crosses the lateral surface of the sternocephalic muscle (musculus sternocephalicus). After draining into the subclavian veins (venae subclaviae), it continues as the brachiocephalic vein (vena brachiocephalica) and enters the cranial vena cava (CrVC, vena cava cranialis), connecting to the right atrium (1). In dogs, veins from the face typically drain into the EJV, with the internal jugular vein (IJV, vena jugularis interna) either absent or present as a small vessel accompanying the carotid artery. In humans, the drainage pattern is different, as the common facial veins from the brain, face, and neck structures drain mainly into the IJV (2).

The significance of the EJV is becoming increasingly evident in veterinary clinical practice. In dogs and cats, the jugular vein becomes the primary choice for blood collection, especially when collecting more than 5 mL of blood, with the EJV being the preferred site when the dog is positioned in a sitting posture (3). Additionally, in the field of veterinary interventional medicine, the right jugular vein in dogs is commonly used as the site for placing vascular introducers during procedures such as balloon valvuloplasty or other transvenous catheter interventions (4). Surgeons must be well-informed about the aspects of the EJV, as a lack of awareness can lead to several complications during neck region surgeries (5). Jugular vein anomalies can significantly influence clinical decision-making concerning vascular access in dogs (6). Despite its importance, to our knowledge, there have been no reports in veterinary medicine focusing on EJV variations in any dog breed. Although anatomical variations of the IJV and EJV have been extensively studied in humans (5, 7), similar investigations in veterinary medicine are limited. Anatomical vascular variations of the IJV in cats (8) and EJV variants in three bulldogs with pulmonary valve stenosis have been documented, including the unilateral absence of the right EJV and bilateral hypoplasia of the EJVs associated with persistent left cranial vena cava (PLCVC, vena cava cranialis sinistra persistens) (9).

In the authors’ experience, EJV variants were more prevalent in Shih Tzu dogs than in other breeds and were often considered incidental findings. Consequently, we hypothesized that the prevalence of EJV variants would be particularly high in Shih Tzu dogs. Additionally, we postulated that anatomical malformations of the EJV and its tributaries could differentiate between EJV anomaly types, offering valuable insights for veterinary clinicians and radiologists. This study aimed to use CT imaging to evaluate the prevalence of anatomic vascular variations of the EJVs in Shih Tzu dogs and to describe the clinical and CT characteristics of these vascular variants.

## 2 Materials and methods

### 2.1 Selection criteria and computed tomographic scanning techniques

This is a retrospective, multicenter, analytical study. The medical imaging records of Shih Tzu dogs that underwent pre- and post-contrast CT examinations of the head, neck, and thorax at the Veterinary Medical Teaching Hospital, Konkuk University, and 10 referral small animal hospitals^1^ were reviewed. All dogs included in this study were examined between 2015 and 2023. In total, 547 CT scans of Shih Tzu dogs were retrospectively evaluated. Both sexes, regardless of height or weight, were included in the study. However, 18 cases were excluded from the PLCVC prevalence analysis because the heart-based region necessary for diagnosing PLCVC was not included in the region of interest (ROI) of the CT examinations.

### 2.2 Data collection and evaluation

Post-contrast CT images were primarily used to analyze the jugular veins and diagnose PLCVC. However, both pre- and post-contrast CT images were assessed. Pre-contrast CT images in which the jugular veins were clearly visible were included in the analysis. The inclusion criterion was the presence of pre- or post-contrast CT images of the neck and thoracic region of Shih Tzu dogs, regardless of clinical signs. When the data were available from the head to the thoracic cavity, the anatomical variations and patterns of related blood vessels were meticulously analyzed. Anatomical variations in the EJVs were evaluated on transverse images at the level of the third to fourth cervical vertebrae around the sternocephalic muscle. Their diameters were measured when both the EJVs and IJVs were visible on a single image. The horizontal and vertical diameters of the right and left EJVs and IJVs were measured and recorded at the third and fourth cervical levels in the axial plane. The presence of PLCVC was evaluated using transverse images at the level of the heart base (cranial vena cava). The Digital Imaging and Communications in Medicine (DICOM) files of CT images were retrieved and reviewed using an image analysis workstation (Radiant^™^, Medixant, Poznan, Poland).

### 2.3 Statistical analysis

Statistical analyses were conducted using commercial software (SPSS 22.0; IBM SPSS Statistics, New York, USA). Descriptive statistics were calculated, and the mean values with standard deviations (SD) were reported. The mean diameters were reported as the mean and 95% confidence intervals (CI). The Kruskal–Wallis test was used to compare the horizontal and vertical diameters of the EJV and IJV among three groups: the normal group, unilateral EJV variants (including hypoplasia and aplasia), and bilateral EJV variants. *Post hoc* adjustments for multiple comparisons were performed using the Mann–Whitney U-test with Bonferroni correction. The correlation between the diameters of the EJV and IJV in the types II unilateral EJV hypoplasia group was evaluated using Pearson’s correlation coefficient. An *R*-value ≥0.7 or ≤ −0.7 was considered strongly correlated, while 0.3 ≤ *R*-value ≤0.7, or − 0.7 ≤ *R*-value ≤ −0.3, was considered moderately correlated. The association between the presence of PLCVC and vascular variations of the EJV was assessed using Fisher’s exact test, and the results were expressed as odds ratios (ORs) with 95% confidence intervals (CI). Sex differences in anomalies were assessed using chi-square analysis. Statistical significance was set at *p* < 0.05.

## 3 Results

CT images from 547 Shih Tzu dogs were evaluated. Abnormalities in the EJV were observed, and when present, various aspects of the vascular drainage through the EJV tributaries were identified, encompassing associated areas of the face and neck. The study included 285 males (52%), 260 females (48%), and 2 unidentified patients.

**Figure 1:**
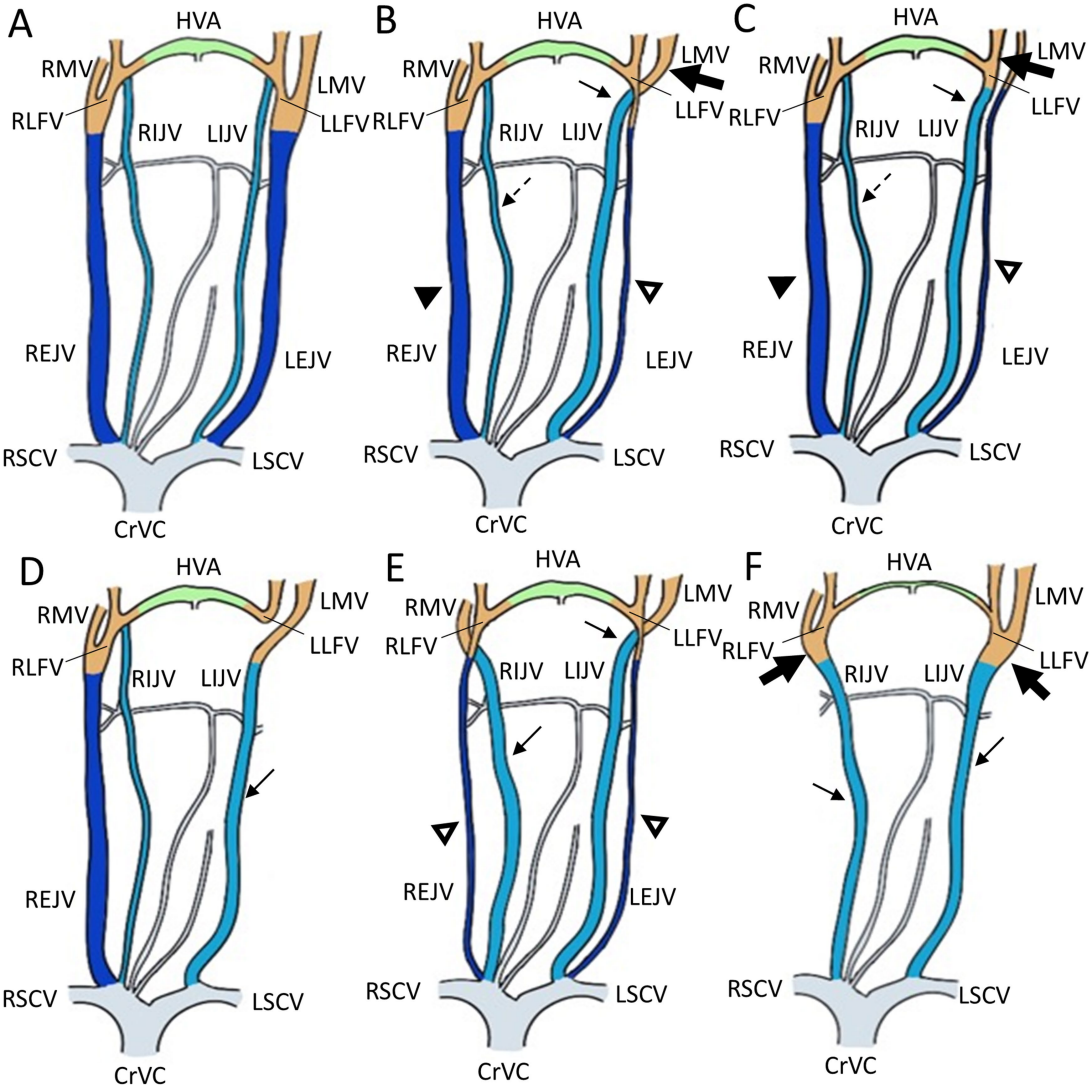
Illustrations of the appearance of the external jugular vein variants in Shih Tzu dogs. **(A)** Schematization of a normal external jugular vein and associated veins: Type I. **(B,C)** Unilateral external jugular vein hypoplasia with two different tributary patterns: Type II. **(B)** The left maxillary vein runs medially (the large black arrow) and directly becomes an enlarged IJV (the black arrow), while the left linguofacial vein joins the hypoplastic left EJV (the open arrowheads). On the contrary, the right maxillary and linguofacial veins join the lateral side of the salivary gland and drain into a normal-sized right EJV (the black arrowheads) with an average-sized right IJV (the dashed arrows). **(C)** The left linguofacial vein runs medially (the large black arrow) and directly becomes an enlarged IJV (the black arrow), while the left maxillary vein joins the hypoplastic left EJV (the open arrowheads). On the contrary, the right maxillary and linguofacial veins join the lateral side of the salivary gland and drain into a normal-sized right EJV (the black arrowheads) with a normal-sized right IJV (the dashed arrows). **(D)** Unilateral external jugular vein aplasia: Type III. Note the left enlarged IJV (the black arrow). **(E)** Bilateral external jugular vein hypoplasia: Type IV. Note the bilateral hypoplastic EJV (the open arrowheads) and enlarged IJV (the black arrow). **(F)** Bilateral external jugular vein aplasia: Type V. Note the convergence of the linguofacial and maxillary veins running medially (the large black arrow) and becoming the enlarged IJV (the black arrow). CrVC, cranial vena cava; RSCV, right subclavian vein; LSCV, left subclavian vein; RMV, right maxillary vein; LMV, left maxillary vein; HVA, hyoid venous arch; RLFV, right linguofacial vein; LLFV, left linguofacial vein; REJV, right external jugular vein; LEJV, left external jugular vein; RIJV, right internal jugular vein; LIJV, left internal jugular vein.

We identified five types of vascular variants of the EJV using CT images (Figure 1): type I: normal type, where EJV morphology and diameter were within the average range when comparing the right and left EJVs; type II: unilateral EJV hypoplasia type, where one EJV appeared significantly narrower than the other upon comparison of their sizes; type III: unilateral EJV aplasia type, where one of the EJVs, either on the right or left side, was absent at its expected location; type IV: bilateral EJV hypoplasia type, where both right and left EJVs were observed to be narrower than the average; and type V: bilateral EJV aplasia type, where neither right nor left EJVs were detected.

**Figure 2:**
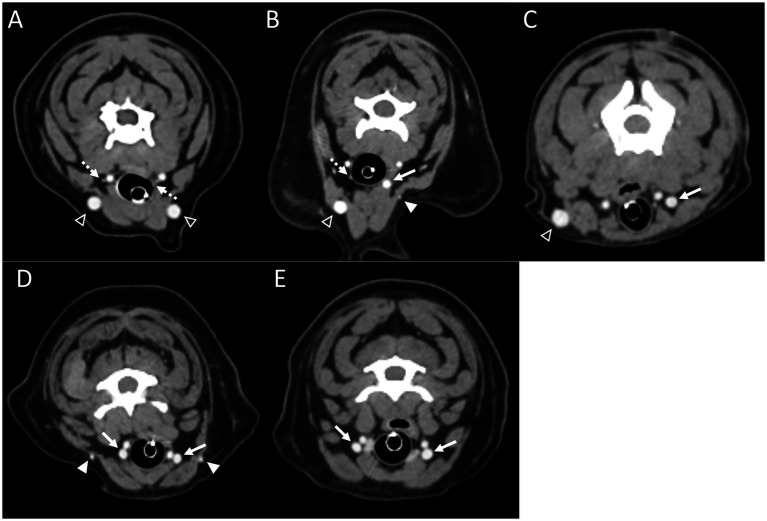
Representative postcontrast CT images of Type I **(A)**, Type II **(B)**, Type III **(C)**, Type IV **(D)**, and Type V **(E)** in transverse images at the level of the 3rd to 4th cervical vertebrae. Note the hypoplastic EJV (the white arrowheads) and enlarged IJV (the white arrow), compared with normal EJV (the open arrowheads) and IJV (the dashed arrows). EJV, external jugular vein; IJV, internal jugular vein.

A total of 119 (21.7%) EJV variants were identified in the CT images. Among these cases, there were 53 male (45%) and 66 female (55%) patients. No significant relationship between sex and the presence of EJV abnormalities was identified, as assessed by Pearson’s chi-squared test (*p* > 0.05). External jugular vein variation types and sidedness are presented in type I pattern, representing a normal formation of the EJV without any hypoplastic morphology, was observed in 428 (78.2%) Shih Tzu dogs. The type II conformation was detected in 46 (8.4%) patients, with 28 in the right EJV and 18 in the left EJV. The type III abnormalities were noted in 41 (7.5%) patients, with 24 in the right EJV and 17 in the left EJV. Type IV anomalies and type V aplasia were observed in 14 (2.6%) and 18 (3.3%) patients, respectively. Among the 87 cases of types II and III cases, hypoplastic or aplastic EJV were present on the right side in 52 cases (59.8%) and on the left side in 35 cases (40.2%) (Table 1). There was no significant difference in the presence of EJV variation between the right and left sides (*p* > 0.05). Representative CT images of the five types of EJV vascular variants in Shih Tzu dogs are shown in Figure 2.

**Table 1:** External jugular vein variation types and sidedness.

EJV variants type	Number of Shih Tzu dogs			
	Total (*n* = 547)	Right (*n* = 52)	Left (*n* = 35)	*p* value
Type I: Normal type	428 (78.2%)			
Type II~V: variants type	119 (21.7%)			
Type II: Unilateral EJV hypoplasia	46 (8.4%)	28	18	0.825
Type III: Unilateral EJV aplasia	41 (7.5%)	24	17	
Type IV: bilateral EJV hypoplasia	14 (2.6%)			
Type V: bilateral EJV aplasia	18 (3.3%)			

*p-value < 0.05 was considered statistically significant in Pearson’s Chi-square test. All data was presented as numbers and percentages. EJV, external jugular vein; PLCVC, persistent left cranial vena cava; CrVC, cranial vena cava.*

**Figure 3:**
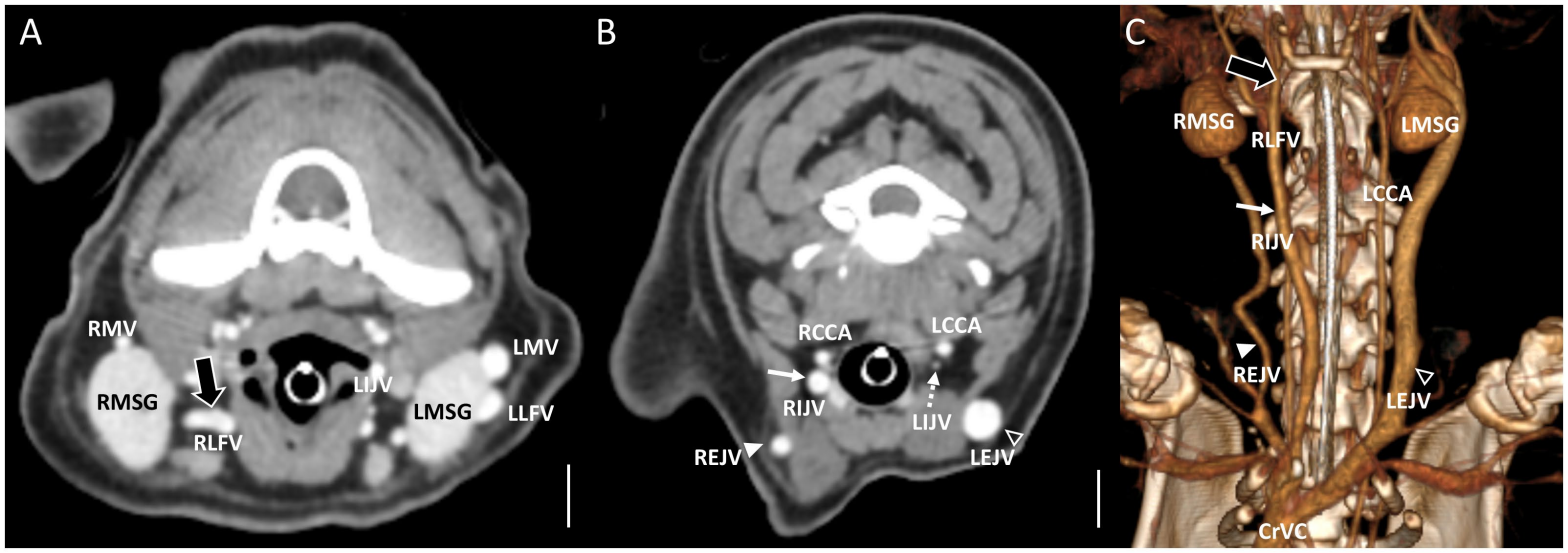
Representative postcontrast CT images of Type II unilateral EJV hypoplasia with tributary patterns in reformatted three-dimensional **(C)** and transverse planes **(A,B)** at the mandibular salivary gland level **(A)** and at the 3rd to 4th cervical level **(B)**. Scale bar equals 1 cm. **(A,B)** The right linguofacial vein runs medially (the large black arrow) and directly becomes an enlarged IJV (the white arrow), while the right maxillary vein joins the hypoplastic right EJV (the white arrowheads). **(B)** On the contrary, the left maxillary vein and linguofacial vein join the lateral side of the salivary gland and drain into a normal-sized left EJV (the open arrowheads) with a normal-sized left IJV (the dashed arrows). EJV, external jugular vein; IJV, internal jugular vein; RCCA, right common carotid artery; LCCA, left common carotid artery; RMSG, right mandibular salivary gland; LMSG, left mandibular salivary gland; RMV, right maxillary vein; LMV, left maxillary vein; RLFV, right linguofacial vein; LLFV, left linguofacial vein; REJV, right external jugular vein; LEJV, left external jugular vein; RIJV, right internal jugular vein; LIJV, left internal jugular vein; CrVC, cranial vena cava.

**Figure 4:**
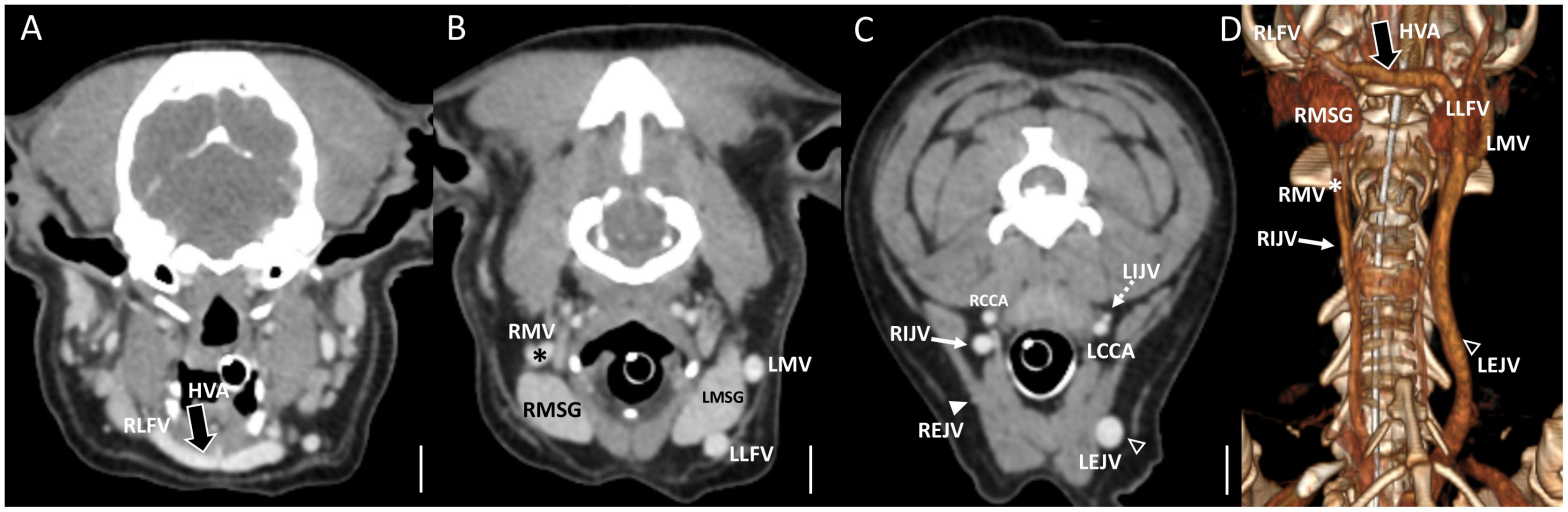
Representative postcontrast CT images of Type II unilateral EJV hypoplasia with tributary patterns in reformatted three-dimensional **(D)** and transverse planes **(A–C)** at the external ear canal level **(A)**, mandibular salivary gland level **(B)**, and at the 3rd to 4th cervical level **(C)**. Scale bar equals 1 cm. **(B,C)** The right maxillary vein runs medially (the asterisks) and directly becomes an enlarged IJV (the white arrow) **(A,D)**, while the right linguofacial vein joins the opposite side EJV (the open arrowheads) through the enlarged hyoid venous arch (the large black arrows). **(C)** On the contrary, the left maxillary and linguofacial veins, connected with the hyoid venous arch, converge at the lateral side of the salivary gland and drain into a normal-sized left EJV (the open arrowheads) with a normal-sized left IJV (the dashed arrows). EJV, external jugular vein; IJV, internal jugular vein; RMSG, right mandibular salivary gland; LMSG, left mandibular salivary gland; RCCA, right common carotid artery; LCCA, left common carotid artery; RMV, right maxillary vein; LMV, left maxillary vein; RLFV, right linguofacial vein; LLFV, left linguofacial vein; REJV, right external jugular vein; LEJV, left external jugular vein; RIJV, right internal jugular vein; LIJV, left internal jugular vein; CrVC, cranial vena cava; HVA, hyoid venous arch.

**Figure 5:**
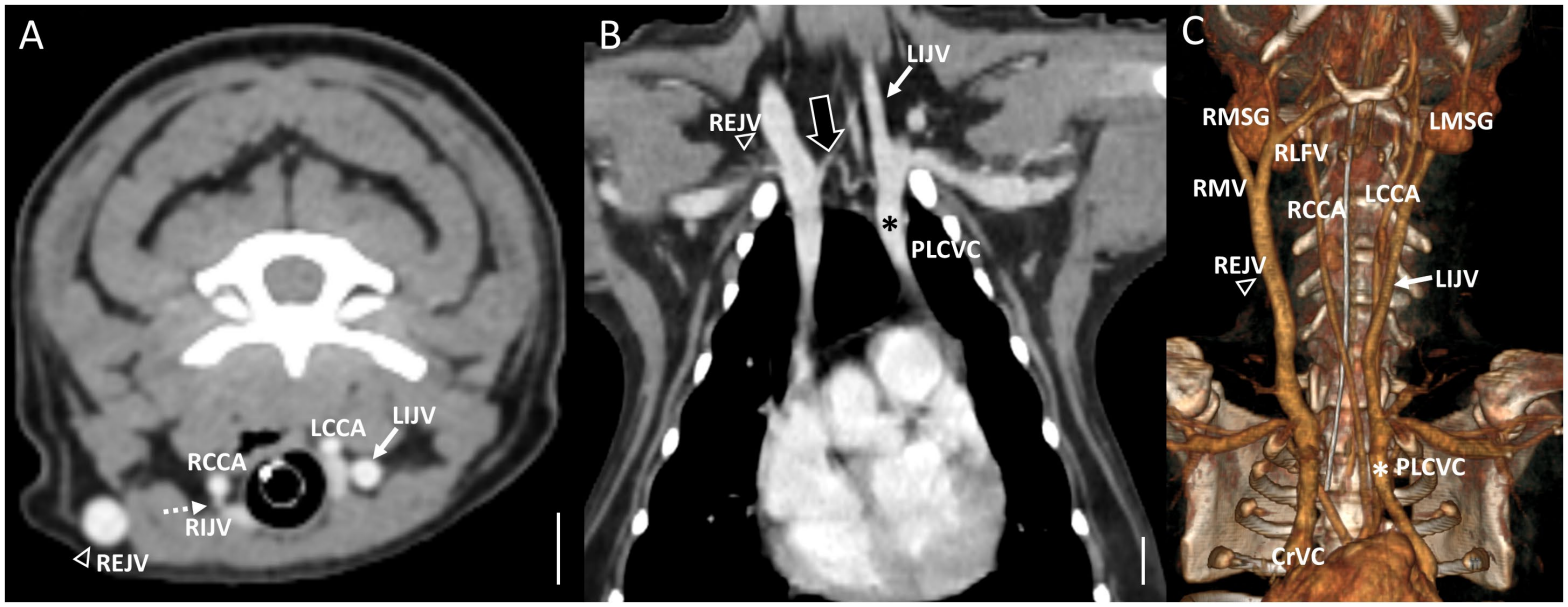
Representative postcontrast CT images of the coexistence of both Type III left side EJV aplasia and PLCVC in reformatted three-dimensional **(C)**, transverse plane **(A)** at the cervical level, and dorsal plane **(B)** at thoracic inlet level. Scale bar equals 1 cm. **(A,C)** The left facial and maxillary veins merged, forming the enlarged IJV (the white arrow), and the left lingual vein drained into the opposite EJV (the open arrowheads) through the hyoid venous arch. **(B)** The enlarged IJV (the white arrows) connected to the left brachiocephalic vein, leading to the formation of the PLCVC (the asterisks) with a bridging vein (the large black arrow) between the PLCVC and CrVC. Note the normal-sized right EJV (the open arrowheads) and right IJV (the dashed arrows). EJV, external jugular vein; IJV, internal jugular vein; RMSG, right mandibular salivary gland; LMSG, left mandibular salivary gland; RCCA, right common carotid artery; LCCA, left common carotid artery; RMV, right maxillary vein; LMV, left maxillary vein; RLFV, right linguofacial vein; LLFV, left linguofacial vein; REJV, right external jugular vein; LEJV, left external jugular vein; RIJV, right internal jugular vein; LIJV, left internal jugular vein; CrVC, cranial vena cava; HVA, hyoid venous arch.

In cases classified as types II–V (Figures 2B,C), there was a tendency for compensation in response to insufficient drainage caused by the hypoplastic or aplastic variants of the EJV, with the IJV assuming this function on the same side. This speculation was based on the significantly enlarged diameter of the IJV compared to its counterpart, which was either very small or not visible under normal circumstances. In cases of bilateral EJV variations (Figures 2D,E), the diameter of the IJV was notably dilated on both sides, and its size often exceeded that of the common carotid artery. Dilation of the IJV observed on the transverse CT image occurred because one of the two tributaries—the maxillary or linguofacial veins—that typically join the lateral side of the salivary gland and drain into the EJV was diverted instead to the medial side of the gland and directly into the IJV (Figure 3). Typically, in most cases, the remaining vein continued as an EJV with a hypoplastic pattern or joined the opposite side of the EJV through the hyoid venous arch (HVA, arcus hyoideus), resulting in hypoplasia or aplasia (Figure 4). In the case of the coexistence of both type III (left-sided EJV aplasia) and PLCVC, the left facial and maxillary veins merged, forming an enlarged IJV, and the left lingual vein drained into the opposite EJV through the HVA. The enlarged IJV was connected to the left brachiocephalic vein (vena brachiocephalica sinistra), leading to PLCVC formation (Figure 5). Within this context, the identification of a bridging vein between the PLCVC and CrVC could have resulted in the classification of PLCVC as type C (9).

**Figure 6:**
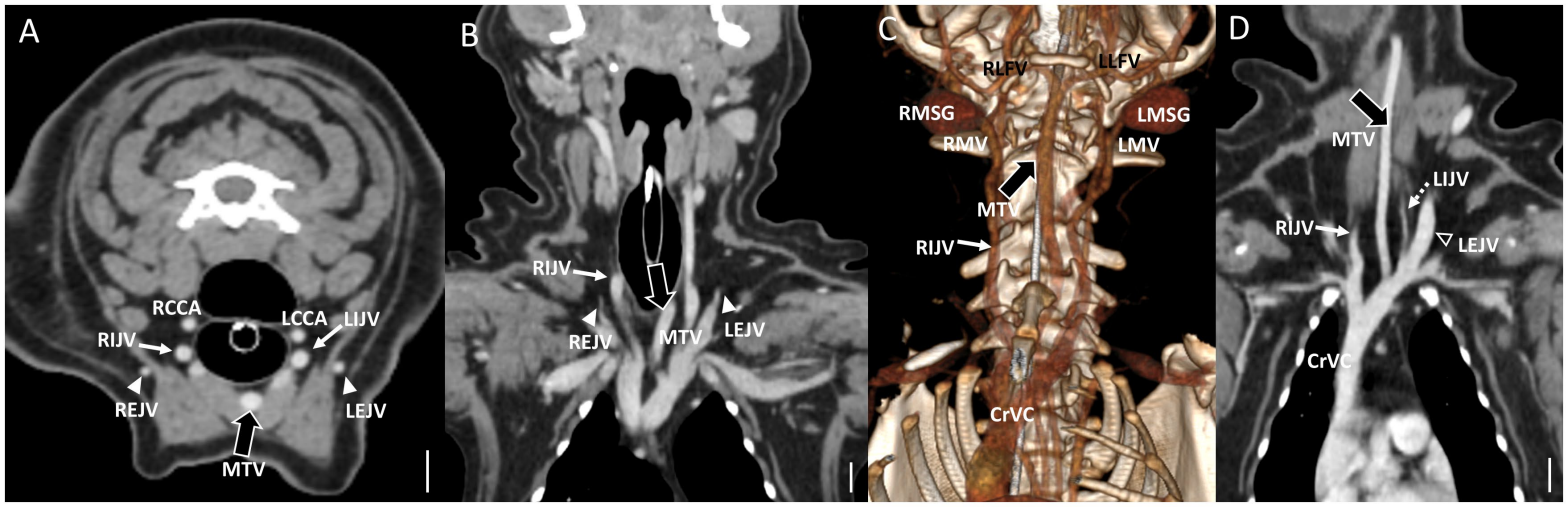
Representative postcontrast CT images of Type IV bilateral EJV hypoplasia with a large vessel passing through the center of the neck in reformatted three-dimensional **(C)**, transverse plane **(A)**, and dorsal plane **(B)** at the cervical level, and Type II unilateral EJV hypoplasia in reformatted dorsal plane **(D)** at thoracic inlet level. Scale bar equals 1 cm. **(A,D)** Note the enlarged “median” thyroid vein (the large black arrows) derived from the caudal thyroid vein, considering its convergence with the left brachiocephalic vein. **(B,C)** Both maxillary veins directly converge into the IJV, whereas both linguofacial veins converge midway and continue their course toward the left brachiocephalic vein. Note the normal-sized left EJV (the open arrowheads) and left IJV (the dashed arrows) compared with the enlarged IJV (the white arrows). EJV, external jugular vein; IJV, internal jugular vein; RMSG, right mandibular salivary gland; LMSG, left mandibular salivary gland; RCCA, right common carotid artery; LCCA, left common carotid artery; RMV, right maxillary vein; LMV, left maxillary vein; RLFV, right linguofacial vein; LLFV, left linguofacial vein; REJV, right external jugular vein; LEJV, left external jugular vein; RIJV, right internal jugular vein; LIJV, left internal jugular vein; CrVC, cranial vena cava; HVA, hyoid venous arch; MTV, median thyroid vein.

In six cases consisting of multiple types (one type II, two type III, two type IV, and one type V), a large vessel passing through the center of the neck was observed. Considering its convergence with the left brachiocephalic vein, this vessel was presumed to be the enlarged “median” thyroid vein (vena thyroidea “media”) derived from the caudal thyroid vein (vena thyroidea caudalis) (Figure 6D). This particular form of the thyroid vein was introduced as a variation in human studies, where many of the variations of the inferior thyroid veins (vena thyroidea inferior) were described (10). Based on this study, it was hypothesized that the “median” thyroid vein in dogs may have originated from the caudal thyroid veins. In the case of (Figures 6A–C) of type IV, both maxillary veins directly converged into the IJV, whereas both linguofacial veins converged midway and continued their course toward the left brachiocephalic vein.

**Figure 7:**
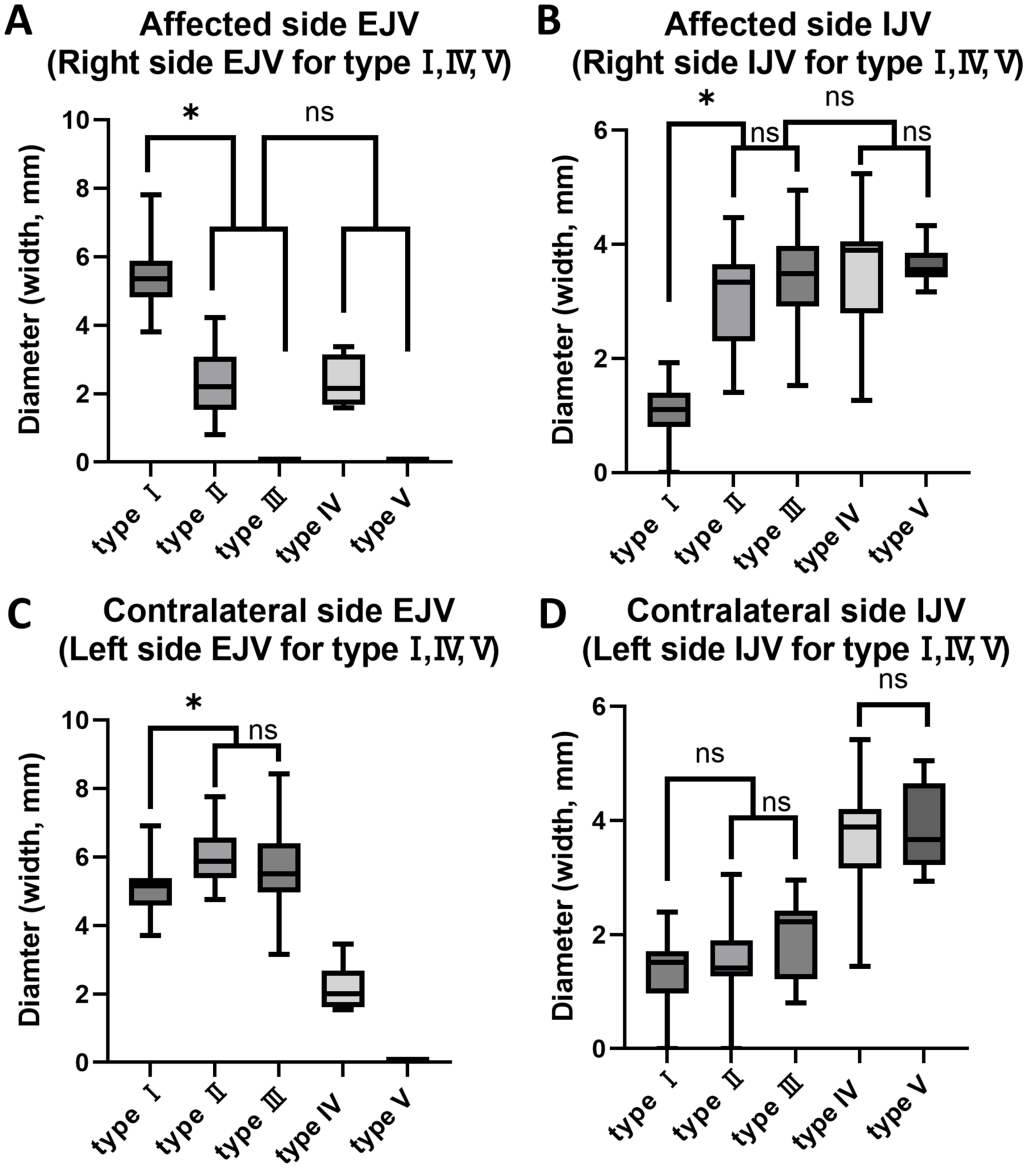
Box and whisker diagrams depicting EJV and IJV measurements for the width of each type in 80 Shih Tzu dogs. Each box represents the interquartile range (IQR), with the horizontal line inside each box indicating the median. The whiskers extend to the minimum and maximum values, representing the full range of the data. **(A)** The hypoplastic or aplastic EJV diameters in the unilateral EJV variants group (types II, III) were significantly narrower than those in the normal group (*p* < 0.001). The affected side EJV of type II, III and the right side EJV in the bilateral EJV deformity group (types IV, V) are not significantly different (*p* > 0.017). **(B)** The affected IJV diameter of types II and III was larger than that in the normal group (*p* < 0.001). The affected side IJV in the unilateral EJV variants group and on the right side in the bilateral EJV deformity group are not significantly different (*p* > 0.017). Between types II and III, and between types IV and V, the affected side IJV/right side IJV did not show significant differences (*p* > 0.017, *p* > 0.017). **(C)** The contralateral EJV diameters in the unilateral EJV variants group were larger than those in the normal group (*p* < 0.001). Between types II and III, the contralateral EJV did not show significant differences (*p* > 0.017). **(D)** The difference in IJV diameter between the normal group and the contralateral IJV in the unilateral EJV variants group was not statistically significant (*p* > 0.017). Between types II and III, and between types IV and V, the contralateral IJV/left side IJV did not show significant differences (*p* > 0.017, *p* > 0.017). EJV, external jugular vein; IJV, internal jugular vein; ns: no significance, *p < 0.001.

The mean measurements of the EJV and IJV diameters for each of the five types are summarized in Table 2. A strong positive correlation (*R* = 0.9) was observed between the vertical and horizontal diameters of the EJV and IJV in the type II unilateral EJV hypoplasia group (*p* < 0.001). A moderate negative correlation (*R* = −0.5) was observed between the hypoplastic EJV and the affected-side IJV (*p* < 0.01). Between types II and III, the diameters of the affected-side IJV, contralateral IJV, and EJV did not differ significantly (*p* > 0.017, *p* > 0.017, and *p* > 0.017, respectively). Between type IV and V, the diameters of the IJVs did not differ significantly (*p* > 0.017, *p* > 0.017). In multiple comparisons of the horizontal and vertical diameters of the EJV and IJV, significant differences were observed between the normal group (type I) and unilateral EJV variants (types II and III), between the unilateral EJV variants (types II and III) and bilateral EJV variants (types IV and V), and between bilateral EJV variants (types IV and V) and the normal group (type I) (*p* < 0.001 for all comparisons). The hypoplastic or aplastic EJV diameters in the unilateral EJV variant group were significantly narrower than those in the normal group (*p* < 0.001), whereas the IJV diameters on the abnormal side in the unilateral EJV variant group were larger than those in the normal group (*p* < 0.001). Additionally, the contralateral EJV diameter was larger than that in the normal group (*p* < 0.001). However, the difference in the IJV diameter between the normal group and the contralateral IJV in the unilateral EJV variant group was not statistically significant (*p* > 0.017). The diameters of the EJV and IJV on the affected side in the unilateral EJV variant group and on the right side in the bilateral EJV deformity group did not significantly differ (*p* > 0.017; Figure 7).

**Table 2:** Descriptive statistics for EJV and IJV diameters in 80 Shih Tzu dogs.

	(mm)	Affected side EJV	Affected side IJV	Contralateral side EJV	Contralateral side IJV
Unilateral hypoplasia (Type II)	Width	2.36 (2.05–2.74)	3.17 (2.85–3.46)	4.86 (4.28–5.40)	2.10 (1.73–2.54)
	Height	2.70 (2.37–3.09)	3.39 (3.07–3.69)	5.27 (4.61–5.94)	2.38 (1.96–2.86)
Unilateral aplasia (Type III)	Width	0^a^	3.44 (3.00–3.84)	5.50 (4.92–6.14)	1.91 (1.56–2.27)
	Height	0^a^	3.85 (3.40–4.26)	5.98 (5.35–6.60)	2.05 (1.69–2.43)

	(mm)	Right side EJV	Right side IJV	Left side EJV	Left side IJV
Normal (Type I)	Width	5.35 (4.96–5.78)	1.02 (0.80–1.24)	4.85 (4.43–5.24)	1.40 (0.99–1.93)
	Height	5.60 (5.18–6.02)	1.17 (0.90–1.44)	5.10 (4.72–5.59)	1.55 (1.12–2.06)
Bilateral hypoplasia (Type IV)	Width	2.33 (1.93–2.74)	3.46 (2.69–4.14)	2.18 (1.83–2.58)	3.65 (2.98–4.27)
	Height	2.63 (2.38–2.91)	3.66 (2.93–4.29)	2.45 (2.14–2.78)	4.12 (3.42–4.75)
Bilateral aplasia (Type V)	Width	0^a^	3.63 (3.39–3.95)	0^a^	3.85 (3.32–4.47)
	Height	0^a^	3.92 (3.58–4.28)	0^a^	4.19 (3.80–4.60)

Vertical and horizontal diameter of the both sides of both the EJV and IJV were measured at the level of C3-C4. All data was presented as median diameter (95%, CI). EJV, external jugular vein; IJV, internal jugular vein. ^a^Not visible on CT image.

Among the 529 Shih Tzu dogs examined with thoracic CT images; 63 cases (11.9%) exhibited PLCVC. All PLCVC patients exhibited a complete type, in which the unatrophied left cranial cardinal vein was connected to the coronary sinus (sinus coronarius) and drained into the right atrium (atrium dextrum). Among the 63 Shih Tzu dogs, the majority demonstrated the common type B. However, variations in complete PLCVC, as detailed in previous studies (9), were observed in four dogs. Two dogs displayed anastomosis via a bridging vein between the right and left CrVCs at the thoracic inlet (type C) (Figure 5), whereas the other two dogs demonstrated PLCVC without a right CrVC (type E). A significant association was found between EJV abnormalities and the presence of PLCVC (*p* value = 0.049, OR = 1.84, 95% CI, 1.03–3.27; Table 3).

**Table 3:** Statistics for the association between EJV variations and the presence of PLCVC.

EJV variants type	PLCVC	CrVC		*p*-value
Type II~V: variants type	20 (17.5%)	94 (82.5%)	114	0.049*
Type I: Normal type	43 (10.4%)	372 (89.6%)	415	
Total (*n* = 529)^a^	63 (11.9%)	466 (88.1%)	529	

*p-value < 0.05 was considered statistically significant in Fisher’s exact test. ^a^18 dogs without thoracic region among 547 dogs were not included. All data was presented as the total number of dogs (n). EJV, external jugular vein; PLCVC, persistent left cranial vena cava; CrVC, cranial vena cava.

During our study, we identified 10 additional dogs of different breeds (3 French bulldogs, 2 Malteses, 1 Japanese spitz, 1 Chihuahua, 1 Boston terrier, 1 Shetland sheepdog, and 1 Shiba inu).

## 4 Discussion

There are two main classification systems for congenital vascular anomalies in humans: the International Society for the Study of Vascular Anomalies (ISSVA) Classification System, revised in 2018, and the Hamburg Classification, developed by Prof. Stefan Belov MD, modified in 2013 (11). The hypoplastic and aplastic anomalies of the EJV identified in this study were expected to be categorized under the ISSVA classification of “Vascular Malformations of Major Named Vessels.” This classification encompassed abnormalities in arteries, veins, and lymphatics, involving anomalies related to their origin, course, number, length, diameter (such as hypoplasia, ectasia, or stenosis), and/or valves (12). The Hamburg classification placed it under “truncular” lesions, indicating that this occurred as the outcome of developmental arrest at a “later” stage of embryogenesis, unlike “extratruncular” form (13). The truncular lesions causing obstruction, such as webs or hypoplasia, may affect venous systems differently depending on their location and severity, leading to hemodynamic compensation through collateral circulation (14). Distinct patterns of compensatory circulation associated with the anomaly types were observed and described in this study.

Enlargement of the IJV on the same side was observed in most cases of the EJV anomalies. A previous study suggested that there might be an inverse correlation between the diameters of the EJV and IJV (15). Similarly, in the current study, a statistically significant moderate negative correlation between these two variables was confirmed. However, four type II cases exhibited no enlargement of the IJV. Among these four cases, three cases showed a maximum diameter of the HVA at the C1-2 level, measuring approximately 4.9, 3.9, and 3.6 mm, indicating a noticeable enlargement similar to the diameter of the left-sided EJV without hypoplasia. Owing to the absence of established norms for the average diameter of the HVA and the lack of a comparative control group for this transversing arch-like vascular structure, assessment of its diameter remains subjective. Upon investigating the diameters of the HVA in type I cases, a range of diameters were observed, from instances where the HVA was entirely absent to those where it measured >2 mm. Similarly, in types II–IV, the diameters varied, ranging from cases where the HVA was completely absent to instances where the diameter was larger than that of the unaffected side’s EJV. However, certain considerations should be taken into account regarding diameter measurements. Factors such as the ventral location of the vascular structures around the hyoid apparatus and the position of sternal recumbency during CT imaging should be considered. This ensures that vascular compression due to the weight of the head may temporarily render them unobservable on imaging, potentially leading to an underestimation or overestimation of their size. Nevertheless, a noticeably larger HVA was observed in the three cases mentioned above than that in the dogs of type I without anomalies. Additionally, in cases where the median thyroid vein was enlarged, despite the absence of established norms for its size, some instances exhibited a significant increase ranging from 1.3 to 5.0 mm. Notably that concomitant enlargement of the IJV was observed in all such cases. Therefore, it could be speculated that the median thyroid vein acts as a supplementary drainage route to the IJV rather than solely draining into both the maxillary and linguofacial veins. In other type II cases, the left EJV did not arise from the brachiocephalic vein; instead, it originated solely from the axillary vein (vena axillaris), extending from the subclavian vein (vena subclavia), and emerged between the cleidocephalic muscle (musculus cleidocephalicus) layers to form the hypoplastic EJV. Among subjects with variants, not a single instance was observed where compensatory enlargement was absent.

To the best of our knowledge, a standardized classification defining the specific size criteria for “hypoplasia” or “aplasia” of specific blood vessels is lacking. These terms were assigned based on a subjective evaluation. In one study, the criteria were arbitrarily established for assessment (16), and we adopted the same criteria for evaluation in our study. The “hypoplastic” EJV was determined by a diameter measuring less than approximately 50% of that in the contralateral vessel on axial images. When evaluating bilateral vessel hypoplasia, despite the absence of a directly comparable contralateral group, a significant narrowing of the vessel diameter was observed in most cases. Numerically, a tendency for a reduction greater than 50% from the average EJV diameter (17) was clearly identified. Aplasia refers to the absence of one or both sides of the EJV from the starting point to the end point of the blood vessel. Likewise, vessel enlargement was defined as a clearly larger vessel diameter than vessels of the same type, and the evaluation of vessel enlargement was also subjective because of similar reasons. In the unilateral cases, while not exactly 50% larger than the contralateral side, it was noticeably larger. In cases classified as types IV and V with bilateral conditions, approximately 1 mm, which is considered a standard diameter for the IJV in dogs, was used as a reference (18).

In cases where the EJV was aplastic, we expected the size of the IJV to be larger than that in cases of a hypoplastic EJV, considering the increased volume of blood that needs to be drained. Contrary to our expectations, the mean diameters of the IJVs did not significantly differ between the hypoplastic EJV group (types II and IV) and the aplastic EJV group (types III and V). Moreover, in contrast to our predictions, no notable difference was observed in the IJV size between unilateral and bilateral occurrences of the EJV variant. However, in cases of unilateral EJV variants, the contralateral EJV had a larger diameter than that in the normal group, which is consistent with our expectations of blood redirection toward the contralateral EJV instead of the anomalous side.

In human cadaver studies on the anatomical variations of the EJV, a shortage of EJV hypoplastic cases was observed (5). However, in our study, EJV hypoplasia was observed in approximately 11% (Type II: 8.4%; Type IV: 2.6%) of the cases, indicating a significant occurrence. Additionally, in a human study, the EJV was absent in 14.2% of cases, all of whom were male, with a majority being on the right side (7). In contrast, our study demonstrated unilateral or bilateral absence of the EJV in 10.3% (type III: 7.5%; type V: 3.3%) of the total cases, with no specific trend observed regarding the left or right side. Upon investigating the etiology of EJV aplasia in dogs, it became evident that this phenomenon differs from that observed in humans. In humans, the absence of the EJV occurs when the retromandibular vein fails to bifurcate into the posterior and anterior branches (19). However, in dogs, the maxillary vein converges with the linguofacial vein, coursing medially to form the IJV, consequently leading to aplasia of the EJV. The canine maxillary vein is characterized by less distinct branching than that observed in humans. Furthermore, in this study, morphological variations such as EJV fenestration or duplication were not observed, contrary to their documented prevalence in numerous human cases (5).

Recently, cardiac interventions, such as balloon dilation of congenitally stenotic valves, coil or device occlusion of anomalous vessels, and extraction of parasites, have been commonly performed in dogs (20). Nearly all cardiac procedures require peripheral vascular access to the circulatory system. In dogs and cats, venous access for interventional procedures is typically achieved through percutaneous methods via the EJV or the femoral vein (vena femoralis). The EJV may be preferred because of its typically larger size than that of the femoral vein. Moreover, the right-side EJV, which offers a more direct path toward the vena cava, is often preferred for vascular interventions (21). Awareness of the EJV variants is crucial for establishing the optimal needle access path during interventional procedures. Although ultrasound guidance for vascular access has been recommended (20), our study proposes the advanced use of CT imaging to detect hypoplasia/aplasia in the EJV, enlargement of the IJV, and the presence of PLCVC. The reason behind this recommendation lies in the possibility of planning vascular access through the IJV when it is enlarged, offering an optimal interventional trajectory rather than relying solely on the EJV (21). In human studies, not knowing the existence of the persistent left superior vena cava (PLSVC) may result in an increased risk of mortality in children undergoing cardiac surgery with cardiopulmonary bypass (22). This study revealed a significant association between EJV variations and the presence of PLCVC, suggesting that it would be helpful to further confirm the presence of PLCVC when EJV abnormalities are detected in similar situations. In dogs, blood samples are commonly collected by venipuncture of the EJV using a syringe and a 20–22-gauge needle (23). There is a risk of damaging nearby muscles or other vessels if jugular venipuncture is attempted without first detecting the presence of variations. Furthermore, the diversity in jugular vein configurations could potentially pose challenges during angiographic and ultrasonographic studies as well as surgical procedures. Therefore, surgeons, clinicians, and radiologists should be accustomed to EJV anomalies and be aware of the critical practical points, particularly in Shih Tzu dogs with a high prevalence of anomalies.

Congenital unilateral or bilateral EJV hypoplasia and aplasia have been reported in various breeds of dogs, including the English bulldogs, French bulldogs, Jack Russel terriers, and Japanese chin dogs (6, 24, 25). In this study, similar variants were observed in breeds other than Shih Tzu dogs, as mentioned earlier in the results. Among the CT images of 150 non-Shih Tzu dog breeds obtained from 2023 to June 2024, EJV variations were observed in 3 dogs (2.0%) (Poodle, French Bulldog, and Shetland Sheepdog), with 2 cases classified as Type II and 1 case as Type IV. These findings are consistent with those of previous unpublished studies (26), which identified aplastic or hypoplastic EJVs just distal to their formation in 3.6% of 1,000 dogs across different breeds. Notably, this study revealed a significantly higher incidence of EJV variation in Shih Tzu dogs (21.7%) than in other breeds. Additionally, PLCVC was observed in three dogs (2.0%) (Poodle and Cocker Spaniel). Therefore, further studies involving a large number of small, medium, and large breeds, or specifically targeting individual breeds, are necessary to determine the prevalence of EJV variants and understand their tendencies.

The retrospective nature of the study resulted in some limitations owing to the non-standardized imaging and patient conditions. The non-standardized contrast medium protocol, CT imaging position, and hydration status may have influenced the circulating blood volume and altered the appearance of the EJV. It is also plausible to consider the possibility of secondary hypoplasia of the EJV caused by jugular venipuncture, which is commonly performed in veterinary clinics.

## 5 Conclusion

In conclusion, the current study describes anatomical variants of the EJV in a sample of Shih Tzu dogs and introduces a novel classification system based on laterality and degree of formation: normal, unilateral hypoplasia, unilateral aplasia, bilateral hypoplasia, and bilateral aplasia. The results of this study indicate that EJV variants in Shih Tzu dogs and compensatory enlargement of tributaries may be more prevalent than previously reported and may be significantly associated with PLCVC. These findings also provide useful criteria for distinguishing anomalous EJV to prevent potential pitfalls in interventional procedures and diagnostic imaging. Additional research is required to ascertain whether the results of this study are applicable to canines of various breeds.

## Data Availability

The data analyzed in this study is subject to the following licenses/restrictions: the datasets used and/or analyzed during the current study are available from the corresponding author by reasonable request. Requests to access these datasets should be directed to jaehwan@konkuk.ac.kr.

## Footnotes

^1^Bundang Leaders Animal Medical Center (AMC), Helix AMC, Ilsan AMC, Saram N AMC, Shine AMC, Dongdaemun VIP AMC, Nell AMC, SD AMC, Jukjun SKY AMC, and Royal AMC.
